# Letter to the Editor

**Published:** 2017

**Authors:** Cosansu Kahraman, Gokhan Vural Mustafa, Akif Cakar Mehmet

**Affiliations:** Training and Research Hospital, Sakarya University,Sakarya, Turkey; Training and Research Hospital, Sakarya University,Sakarya, Turkey; Training and Research Hospital, Sakarya University,Sakarya, Turkey

Dear Sir

We read the article titled ‘Relationship between coronarytortuosity and plateletcrit coronary tortuosity and plateletcrit’by Cerit et al., published online in the Cardiovascular Journal ofAfrica on 25 April 2017, with great interest (see page 385 in thisissue). However, we have some comments regarding this study.

Although it is a relatively common finding in coronary angiography, little is known about the importance of coronary tortuosity (CorT). As mentioned in the article, clinical studies have demonstrated that CorT may be related to aging, hypertension, atherosclerosis and diabetes mellitus.^1^,^2^

In this study, there was a significant difference between the groups regarding hypertension, diabetes mellitus and aging. For this reason, it was to be expected that CorT would be higher in the group with these risk factors. High blood pressure itself initiates a chain of events in vessel walls in the form of oxidative stress, inflammation and endothelial dysfunction.^3^

Similarly, in diabetes, Herder et al. found an increased level of inflammatory reactions and their components, including inflammatory parameters.^4^ In previous studies, PCT, MPV, NLR and PLR were found to be markers of inflammatory status.

In conclusion, we would have expected higher PCT, MPV, NLR and PLR values in the CorT group. It would have been preferable if there were no differences between the two groups in terms of hypertension, diabetes mellitus and aging. Then PCT, MPV, NLR and PLR would have provided more accurate information about the ability of CorT to predict disease. Future studies should be directed towards larger randomised trials with more emphasis on long-term clinical endpoints.
